# Dataset on share issuance, abnormal returns and market timing in the Brazilian stock market

**DOI:** 10.1016/j.dib.2019.104251

**Published:** 2019-07-14

**Authors:** M.C. Gomes, V.M. Magnani, T. Albanez, M.R. Valle

**Affiliations:** University of São Paulo, Brazil

**Keywords:** Capital structure, Financing decision, Share issuance, Abnormal returns, Brazilian stock market

## Abstract

This article presents a dataset to investigate the determinants of firms’ decision for primary share issuance and the effects of market timing on primary share issues in the Brazilian stock market. The data refer to Brazilian nonfinancial firms that issued primary shares (IPOs and SEOs) in the 2004–2015 period. The data were gathered from the online bases of Economatica^®^ and the São Paulo Securities, Commodities and Futures Exchange (BM&FBovespa). The final sample was composed of 123 firms and 165 primary share issues: 97 initial public offerings and 68 follow-on offerings. The dataset was developed to support a model that captures market timing behavior through cumulative abnormal returns and shows the effects of this behavior on the amount of proceeds raised. The dataset contains subsamples and different analysis time windows, processed and unprocessed data. Researchers can use the dataset for future research and comparisons with other markets and models. The related research article using part of the current dataset was published under the following title: “Effects of market timing on primary share issues in the Brazilian capital market” (Gomes et al., 2019).

**Table:** Specifications table

Subject area	Economics
More specific subject area	Finance
Type of data	XLS files and tables in the article.
How data was acquired	The data on the firms share issues in the Brazilian market between 2004 and 2015 were gathered from the online database of the São Paulo Securities, Commodities, and Futures Exchange (BM&FBovespa) (http://www.bmfbovespa.com.br). The quarterly data for calculation of the control variables used in the models were obtained from the Economatica^®^, a private database. First, the data was collected and processed in Excel spreadsheets. Afterward, the statistical package STATA was used for data analysis.
Data format	Processed, analyzed.
Experimental factors	We excluded banks, insurers, insurance brokers, and investment funds due to the particular characteristics of their capital structure. Then, like other authors, we banned firms that presented the following conditions: (1) total assets worth less than R$ 10 million; (2) missing accounting information in the main database used (Economatica^®^) that impairs the analysis; (3) book leverage higher than 1 (or 100%); and (4) market-to-book ratio higher than 10.
Experimental features	We used descriptive statistics and linear regression models to analyze the relationship between market timing and abnormal returns. The statistical package STATA was used for data analysis. We lagged the control variables by one period to minimize multicollinearity and problems of heteroscedasticity. We also used robust variance/covariance matrices of the parameters (White's correction).
Data source location	Laboratory of Finance and Risk (RiskFinLab), University of São Paulo (USP), School of Economics, Business and Accounting (FEA), Department of Accounting, São Paulo, Brazil.
Data accessibility	Data included in this article.
Related research article	A relevant research article to this dataset is Gomes MC, Magnani VM, Albanez T & Valle MR, Effects of market timing on primary share issues in the Brazilian capital market. *The North-American Journal of Economics and Finance*, 49, 361–377 [1].

**Table:** 

**Value of the data** • The dataset provided information about firms that carried out IPOs and follow-on offerings in the Brazilian market between 2004 and 2015, composed of 123 companies and 165 primary share issues: 97 initial public offerings and 68 follow-on offerings. This dataset is useful to investigate the determinants and consequences of firms’ decisions for primary share issuance. • The dataset contains cross-sectional firm-level data such as the amount of capital raised, the number of shares issued, the price per share, firm size, tangibility, profitability, book leverage, market-to-book, and cumulative abnormal returns. Consequently, the dataset is particularly useful for those who study the effects of market timing on firms financing decisions. The data support, for example, a model that captures market timing behavior through cumulative abnormal returns and the impact of this behavior on the amount of proceeds raised, and can be applied and interpreted in other markets. • The dataset contains subsamples and different analysis time windows, indicating the dimension and characteristics of the Brazilian context, and may inspire researchers to explore developing issues related to the phenomenon of market timing behavior and its effects.

## 1 Data

The data on the firms that carried out IPOs and follow-on offerings in the Brazilian market between 2004 and 2015 were gathered from the online database of the São Paulo Securities, Commodities and Futures Exchange (BM&FBovespa) (http://www.bmfbovespa.com.br). In this interval, there were 222 primary stock issues: 85 follow-on offerings and 137 IPOs. The final sample of firms came from a wide range of economic sectors.

To compose the final sample, we excluded banks, insurers, insurance brokers, and investment funds, due to the particular characteristics of their capital structure. Then, like other authors, we excluded firms that presented the following conditions: (1) total assets worth less than R$ 10 million; (2) missing accounting information in the main database used (Economatica^®^) that impairs the analysis; (3) book leverage higher than 1 (or 100%); and (4) market-to-book ratio greater than 10. The final sample was composed of 123 companies and 165 primary share issues: 97 initial public offerings and 68 follow-on offerings. The quarterly data for calculation of the control variables, such as firm size, tangibility, profitability, book leverage, and market-to-book, were obtained from the Economatica^®^ database.

First, the data was collected and processed in Excel spreadsheets. Afterward, the statistical package STATA was used for data analysis. Table 1 summarizes the calculation of each variable. Table 2 presents the average values of the dependent variables for the sample with 68 follow-on offerings, and Table 5 shows the average values for the complete sample. Table 3, Table 4, Table 6, Table 7, Table 8, Table 9, Table 10, Table 11, Table 12 present the data of cross-sectional regressions for different model specifications.

**Table 1:** Description of dataset variables.

Variables	Abbrev.	Definition/Calculation
**Dependent Variables**		
Amount of capital raised	*Cap1*	Capital raised/Total assets
Number of shares issued	*Cap2*	No. of shares offered/No. of shares outstanding
Price per share	*Cap3*	Price per share offered/Book value of the share
**Explanatory variables**		
Cumulative abnormal return before share issue	*CAR1*_*t*_	Obtained using monthly benchmark-adjusted returns before share issue, for three different windows (t = 6, 12 and 18 months)
Cumulative abnormal return after share issue	*CAR2*_*t*_	Obtained using monthly benchmark-adjusted returns after share issue, for three different windows (t = 6, 12 and 18 months)
Dummy for cumulative abnormal return before share issue	*D_CAR1*_*t*_	=1 for firms with positive CAR1 and zero otherwise, for each of the three different windows (t = 6, 12 and 18 months)
		
Dummy for cumulative abnormal return after share issue	*D_CAR2*_*t*_	=1 for firms with negative CAR2 and zero otherwise, for each of the three different windows (t = 6, 12 and 18 months)
Dummy for cumulative abnormal return before and after share issue	*D_CAR1*_t_*D_CAR2_*t*_	=1 for firms with both D_CAR1 and D_CAR2 are 1, and zero otherwise, for each of the three different windows (t = 6, 12 and 18 months)
**Control variables**		
Firm size	*Size*	ln(total assets)
Tangibility	*Tang*	(Fixed assets – Reval. Res.)/Total assets
Profitability	*Prof*	EBITDA/Total assets
Book leverage	*Lev*	DL/Total assets
Market-to-book	*M/B*	Market value of assets/Book value of assets
Sectorial market-to-book	*M/Bs*	Median of the M/B ratios of sector i at time t

Notes: *Ln*, natural logarithm; *Reval. Res.*, revaluation reserve; *EBITDA*, earnings before interest, taxes, depreciation and amortization; *DL*, debt liabilities, composed of short- and long-term loans, bonds and commercial lease obligations.

**Table 2:** **Mean values and difference of means tests: sample with 68 follow-on offerings in the analysis of the D_CAR1**_**t**_. This table presents the average values of the dependent variables, segregated in the two groups of observations obtained by the measure *D_CAR1*_*t*_, along with the difference of means test (Mann-Whitney). Only the sample with 68 follow-on offerings was used in the analysis of the variable *D_CAR1*_*t*_ (*t* = 6, 12 and 18 months).

Variable	N	Statistic	Cap1	Cap2	Cap3
D_CAR1_6_= 1	n = 47	Mean	0.169	0.171	1.212
		Standard Deviation	0.146	0.146	0.146
D_CAR1_6_= 0	n = 21	Mean	0.138	0.258	0.745
		Standard Deviation	0.218	0.218	0.218
Mann-Whitney Test (z)			(0.118)	(0.332)	(1.778)*
D_CAR1_12_= 1	n = 54	Mean	0.171	0.187	1.153
		Standard Deviation	0.136	0.136	0.136
D_CAR1_12_= 0	n = 14	Mean	0.116	0.239	0.738
		Standard Deviation	0.267	0.267	0.267
Mann-Whitney Test (z)			(0.180)	(0.174)	(1.385)
D_CAR1_18_= 1	n = 58	Mean	0.167	0.181	1.158
		Standard Deviation	0.131	0.131	0.131
D_CAR1_18_= 0	n = 10	Mean	0.113	0.291	0.543
		Standard Deviation	0.316	0.316	0.316
Mann-Whitney Test (z)			(0.157)	(0.321)	(1.797)*

Notes: *Cap1* is the amount of capital raised; *Cap2* is the number of shares issued; *Cap3* is the price per share. *D_CAR1*_*t*_ = 1 for positive cumulative abnormal return before the share issue and 0 otherwise, (*t* = 6, 12 and 18 months). For rejection of the null hypothesis of no difference in means: ***significance at 1%; ** significance at 5%; * significance at 10%.

**Table 3:** **Effect of abnormal returns on primary share issues: sample with 68 follow-on offerings, using the variable sectorial market-to-book ratio (M/Bs)**. This table presents the data of cross-sectional regression. Only the sample with 68 follow-on offerings was used in the analysis of the variable *CAR1*_*t*_ (*t* = 6, 12 and 18 months). The variables are those contained in Model 1, including the sectorial M/B (M/Bs) in place of the firm M/B, where *Y*_*ij*_ denotes the dependent variables *j Cap1*, *Cap2* and *Cap3*. Model 1.$Y_{ij}=\alpha +\beta_{1}\;{\left(CAR1\right)}_{i}+\beta_{2}\;{\left(M/Bs\right)}_{i}+\beta_{3}\;{\left(Tang\right)}_{i}+\;\beta_{4}\;{\left(Prof\right)}_{i}+\;\beta_{5}\;{\left(Size\right)}_{i}+\beta_{6\;}{\left(Lev\right)}_{i}+u_{i}$.

		6 months			12 months			18 months		
		Cap1	Cap2	Cap3	Cap1	Cap2	Cap3	Cap1	Cap2	Cap3
CAR1_6_		0.093	0.032	0.014	–	–	–	–	–	–
		(0.004)***	(0.497)	(0.921)	–	–	–	–	–	–
CAR1_12_		–	–	–	0.075	0.016	0.062	–	–	–
		–	–	–	(0.006)***	(0.716)	(0.589)	–	–	–
CAR1_18_		–	–	–	–	–	–	0.027	−0.018	0.023
		–	–	–	–	–	–	(0.355)	(0.578)	(0.791)
M/Bs		0.026	−0.031	0.599	0.024	−0.032	0.601	0.018	−0.031	0.597
		(0.241)	(0.250)	(0.000)***	(0.263)	(0.222)	(0.000)***	(0.362)	(0.211)	(0.000)***
Tang		0.083	0.101	0.067	0.087	0.103	0.066	0.090	0.103	0.069
		(0.018)**	(0.232)	(0.748)	(0.016)**	(0.218)	(0.755)	(0.015)**	(0.236)	(0.744)
Prof		−0.252	−0.785	1.819	−0.253	−0.777	1.774	−0.254	−0.715	1.768
		(0.302)	(0.044)**	(0.267)	(0.320)	(0.043)**	(0.288)	(0.297)	(0.066)*	(0.291)
Size		−0.019	0.006	−0.090	−0.018	0.005	−0.081	−0.028	−0.002	−0.088
		(0.054)*	(0.697)	(0.017)**	(0.069)*	(0.753)	(0.039)**	(0.012)**	(0.891)	(0.022)**
Lev		−0.107	0.128	−1.165	−0.130	0.116	−1.148	−0.148	0.100	−1.161
		(0.116)	(0.165)	(0.004)***	(0.049)**	(0.209)	(0.004)***	(0.036)**	(0.280)	(0.004)***
Const		0.426	0.123	1.777	0.412	0.153	1.599	0.588	0.267	1.740
		(0.013)**	(0.638)	(0.010)***	(0.016)**	(0.525)	(0.019)**	(0.002)***	(0.234)	(0.010)***
N		68	68	68	68	68	68	68	68	68
Prob > F	0.000***	0.078*	0.000***	0.000***	0.092*	0.000***	0.000***	0.084*	0.000***	
R^2^	0.5129357	0.165	0.574	0.496	0.159	0.576	0.392	0.162	0.575	
Estimation with robust matrix	Yes	Yes	Yes	Yes	Yes	Yes	Yes	Yes	Yes	
**Breusch-Pagan test**										
Chi^2^(1)	7.21	14.88	5.43	6.88	13.22	5.69	14.1	11.2	5.75	
Prob > Chi^2^	0.0072***	0.000***	0.020**	0.009***	0.000***	0.017**	0.000***	0.001***	0.0165**	

Notes: *Cap1* is the amount of capital raised; *Cap2* is the number of shares issued; *Cap3* is the price per share. *CAR1*_*t*_ is the cumulative abnormal return before share issue, where *t* = 6, 12 and 18 months. *M/Bs* is sectorial market-to-book ratio; *Tang* is tangibility; *Prof* is profitability; *Size* is firm size; and *Lev* is book leverage. The variables *Tang*, *Prof*, *Size* and *Lev* were lagged by one quarter. We assumed significance of 5% in the Breusch-Pagan test for heteroscedasticity, i.e., when H_0_: homoscedasticity is rejected. The regression was performed by robust standard errors to correct the standard deviation for the possible presence of heteroscedasticity (White's correction). The table presents the linear coefficient of each explanatory variable followed by the p-value in parentheses. For rejection of the null hypothesis of coefficient equal to zero: ***significance at 1%; ** significance at 5%; * significance at 10%.

**Table 4:** **Effect of abnormal returns on primary share issues: sample with 68 follow-on offerings, without the variable market-to-book ratio (M/B)**. This table presents the data of cross-sectional regression. Only the sample with 68 follow-on offerings was used in the analysis of the variable *CAR1*_*t*_ (*t* = 6, 12 and 18 months). The variables are those contained in Model 1, without the variable market-to-book ratio (M/B), where *Y*_*ij*_ denotes the dependent variables *j Cap1*, *Cap2* and *Cap3*. Model 1.$Y_{ij}=\alpha +\beta_{1}\;{\left(CAR1\right)}_{i}+\beta_{2}\;{\left(Tang\right)}_{i}+\;\beta_{3}\;{\left(Prof\right)}_{i}+\;\beta_{4}\;{\left(Size\right)}_{i}+\beta_{5\;}{\left(Lev\right)}_{i}+u_{i}$.

	6 months			12 months			18 months		
	Cap1	Cap2	Cap3	Cap1	Cap2	Cap3	Cap1	Cap2	Cap3
CAR1_6_	0.092	0.034	−0.029	–	–	–	–	–	–
	(0.007)***	(0.451)	(0.847)	–	–	–	–	–	–
CAR1_12_	–	–	–	0.074	0.017	0.036	–	–	–
	–	–	–	(0.000)***	(0.687)	(0.788)	–	–	–
CAR1_18_	–	–	–	–	–	–	0.028	−0.020	0.052
	–	–	–	–	–	–	(0.331)	(0.538)	(0.566)
Tang	0.096	0.085	0.385	0.100	0.086	0.383	0.100	0.086	0.384
	(0.006)***	(0.320)	(0.175)	(0.018)**	(0.305)	(0.176)	(0.008)***	(0.317)	(0.175)
Prof	−0.086	−0.983	5.706	−0.098	−0.981	5.674	−0.138	−0.910	5.535
	(0.657)	(0.003)***	(0.005)***	(0.661)	(0.003)***	(0.006)***	(0.513)	(0.009)***	(0.012)**
Size	−0.023	0.011	−0.185	−0.022	0.010	−0.174	−0.031	0.002	−0.170
	(0.017)**	(0.433)	(0.001)***	(0.005)***	(0.464)	(0.002)***	(0.003)***	(0.844)	(0.001)***
Lev	−0.128	0.153	−1.658	−0.149	0.141	−1.628	−0.162	0.123	−1.612
	(0.065)*	(0.079)*	(0.001)***	(0.032)**	(0.110)	(0.001)***	(0.028)**	(0.162)	(0.001)***
Const	0.520	0.010	3.995	0.499	0.037	3.803	0.650	0.164	3.741
	(0.001)***	(0.962)	(0.000)***	(0.000)***	(0.849)	(0.000)***	(0.000)***	(0.363)	(0.000)***
N	68	68	68	68	68	68	68	68	68
Prob > F	0.000***	0.060*	0.000***	0.000***	0.073*	0.000***	0.000***	0.065*	0.000***
R^2^	0.498	0.154	0.416	0.483	0.147	0.416	0.384	0.151	0.418
Estimation with robust matrix	Yes	Yes	Yes	No	Yes	Yes	Yes	Yes	Yes
**Breusch-Pagan test**									
Chi^2^(1)	4.06	20.21	6.81	3.77	18.29	7.41	10.16	13.56	7.88
Prob > Chi^2^	0.044**	0.000***	0.009***	0.052*	0.000***	0.007***	0.001***	0.000***	0.005***

Notes: *Cap1* is the amount of capital raised; *Cap2* is the number of shares issued; *Cap3* is the price per share. *CAR1*_*t*_ is cumulative abnormal return before share issue, where *t* = 6, 12 and 18 months. *Tang* is tangibility; *Prof* is profitability; *Size* is firm size; and *Lev* is book leverage. The variables *Tang*, *Prof*, *Size* and *Lev* were lagged by one quarter. We assumed significance of 5% in the Breusch-Pagan test for heteroscedasticity, i.e., when H_0_: homoscedasticity is rejected. The regression was performed by robust standard errors to correct the standard deviation for the possible presence of heteroscedasticity (White's correction). The table presents the linear coefficient of each explanatory variable followed by the p-value in parentheses. For rejection of the null hypothesis of coefficient equal to zero: ***significance at 1%; ** significance at 5%; * significance at 10%.

**Table 5:** **Mean values and difference of means tests: total sample (IPOs and follow-on offerings) in the analysis of D_CAR2**_**t**_. This table presents the average values of the dependent variables, segregated in the two groups of observations obtained by the measure *D_CAR2*_*t*_, along with the difference of means test (Mann-Whitney). The complete sample was used in the analysis of the variable D_*CAR2*_*t*_ (*t* = 6, 12 and 18 months).

Variable	N	Statistic	Cap1	Cap2	Cap3
D_CAR2_6_ = 1	n = 80	Mean	0,348	0,259	1,538
		Standard Deviation	0,112	0,112	0,112
D_CAR2_6_ = 0	n = 85	Mean	0,279	0,228	1,313
		Standard Deviation	0,108	0,108	0,108
Mann-Whitney Test (z)			(0,445)	(0.198)	(1.441)*
D_CAR2_12_ = 1	n = 84	Mean	0,349	0,251	1,525
		Standard Deviation	0,109	0,109	0,109
D_CAR2_12_ = 0	n = 81	Mean	0,275	0,234	1,315
		Standard Deviation	0,111	0,111	0,111
Mann-Whitney Test (z)			(0,478)	(0.109)	(1.349)
D_CAR2_18_ = 1	n = 82	Mean	0,364	0,257	1,611
		Standard Deviation	0,110	0,110	0,110
D_CAR2_18_ = 0	n = 83	Mean	0,262	0,229	1,236
		Standard Deviation	0,110	0,110	0,110
Mann-Whitney Test (z)			(0,652)	(0.180)	(2.410)**

Notes: *Cap1* is the amount of capital raised; *Cap2* is the number of shares issued; *Cap3* is the price per share. *D_CAR2*_*t*_ = 1 for negative cumulative abnormal return after the share issue and 0 otherwise, *(t* = 6, 12 and 18 months). For rejection of the null hypothesis of no difference in means: ***significance at 1%; ** significance at 5%; * significance at 10%.

**Table 6:** **Effect of abnormal returns on primary share issues: total sample (IPOs and follow-on offerings), using the variable *CAR2***_***t***_**and sectorial market-to-book ratio (M/Bs)**. This table presents the data of cross-sectional regression. The complete sample was used in the analysis of the variable *CAR2*_*t*_ (*t* = 6, 12 and 18 months). The variables are those contained in Model 2, including the sectorial M/B (M/Bs) in place of the firm M/B, where *Y*_*ij*_ denotes the dependent variables *j Cap1*, *Cap2* and *Cap3*. Model 2.$Y_{ij}=\alpha +\beta_{1}\;{\left(CAR2\right)}_{i}+\beta_{2}\;{\left(M/Bs\right)}_{i}+\beta_{3}\;{\left(Tang\right)}_{i}+\;\beta_{4}\;{\left(Prof\right)}_{i}+\;\beta_{5}\;{\left(Size\right)}_{i}+\beta_{6\;}{\left(Lev\right)}_{i}+u_{i}$.

	6 months			12 months			18 months		
	Cap1	Cap2	Cap3	Cap1	Cap2	Cap3	Cap1	Cap2	Cap3
CAR2_6_	−0.023	−0.001	−0.381	–	–	–	–	–	–
	(0.601)	(0.985)	(0.083)*	–	–	–	–	–	–
CAR2_12_	–	–	–	−0.058	−0.031	−0.103	–	–	–
	–	–	–	(0.026)**	(0.335)	(0.429)	–	–	–
CAR2_18_	–	–	–	–	–	–	−0.034	−0.031	−0.006
	–	–	–	–	–	–	(0.089)*	(0.155)	(0.955)
M/Bs	0.016	−0.045	0.578	0.013	−0.046	0.569	0.012	−0.048	0.574
	(0.455)	(0.025)**	(0.000)***	(0.541)	(0.019)**	(0.000)***	(0.549)	(0.017)**	(0.000)***
Tang	0.042	0.021	0.469	0.040	0.019	0.464	0.037	0.017	0.468
	(0.426)	(0.674)	(0.074)*	(0.445)	(0.691)	(0.079)*	(0.475)	(0.738)	(0.078)*
Prof	−0.381	−0.295	0.355	−0.299	−0.246	0.325	−0.335	−0.244	0.168
	(0.014)**	(0.009)***	(0.641)	(0.056)*	(0.044)**	(0.680)	(0.032)**	(0.032)**	(0.829)
Size	−0.105	−0.030	−0.208	−0.104	−0.029	−0.209	−0.104	−0.028	−0.211
	(0.000)***	(0.006)***	(0.000)***	(0.000)***	(0.007)***	(0.000)***	(0.000)***	(0.009)***	(0.000)***
Lev	−0.080	0.002	−0.794	−0.091	−0.007	−0.736	−0.082	−0.005	−0.707
	(0.259)	(0.980)	(0.023)**	(0.188)	(0.911)	(0.036)**	(0.237)	(0.937)	(0.043)**
Const	1.819	0.754	3.491	1.806	0.747	3.490	1.799	0.735	3.512
	(0.000)***	(0.000)***	(0.000)***	(0.000)***	(0.000)***	(0.000)***	(0.000)***	(0.000)***	(0.000)***
N	165	165	165	165	165	165	165	165	165
Prob > F	0.000***	0.000***	0.003***	0.000***	0.000***	0.004***	0.000***	0.000***	0.003***
R^2^	0.588	0.112	0.460	0.600	0.121	0.451	0.595	0.127	0.449
Estimation with robust matrix	No	Yes	No	No	Yes	No	No	Yes	No
**Breusch-Pagan test**									
Chi^2^(1)	1.54	129.51	0.27	1.68	129.25	0.98	2.14	18.8	0.14
Prob > Chi^2^	0.214	0.000***	0.604	0.195	0.000***	0.322	0.143	0.000***	0.704

Notes: *Cap1* is the amount of capital raised; *Cap2* is the number of shares issued; *Cap3* is the price per share. *CAR2*_*t*_ is accumulative abnormal return after share issue, where *t* = 6, 12 and 18 months. *M/Bs* is sectorial market-to-book ratio; *Tang* is tangibility; *Prof* is profitability; *Size* is firm size; and *Lev* is book leverage. The variables *Tang*, *Prof*, *Size* and *Lev* were lagged by one quarter. We assumed significance of 5% in the Breusch-Pagan test for heteroscedasticity, i.e., when H_0_: homoscedasticity is rejected. The regression was performed by robust standard errors to correct the standard deviation for the possible presence of heteroscedasticity (White's correction). The table presents the linear coefficient of each explanatory variable followed by the p-value in parentheses. For rejection of the null hypothesis of coefficient equal to zero: ***significance at 1%; ** significance at 5%; * significance at 10%.

**Table 7:** **Effect of abnormal returns on primary share issues: total sample (IPOs and follow-on offerings), using the variable *CAR2***_***t***_**, without the variable market-to-book ratio (M/B)**. This table presents the data of cross-sectional regression. The complete sample was used in the analysis of the variable *CAR2*_*t*_ (*t* = 6, 12 and 18 months). The variables are those contained in Model 2, without the variable market-to-book ratio (M/B), where *Y*_*ij*_ denotes the dependent variables *j Cap1*, *Cap2* and *Cap3*. Model 2.$Y_{ij}=\alpha +\beta_{1}\;{\left(CAR2\right)}_{i}+\;\beta_{2}\;{\left(Tang\right)}_{i}+\;\beta_{3}\;{\left(Prof\right)}_{i}+\;\beta_{4}\;{\left(Size\right)}_{i}+\beta_{5\;}{\left(Lev\right)}_{i}+u_{i}$.

	6 months			12 months			18 months		
	Cap1	Cap2	Cap3	Cap1	Cap2	Cap3	Cap1	Cap2	Cap3
CAR2_6_	−0.022	−0.003	−0.354	–	–	–	–	–	–
	(0.612)	(0.948)	(0.139)	–	–	–	–	–	–
CAR2_12_	–	–	–	−0.059	−0.027	−0.146	–	–	–
	–	–	–	(0.023)**	(0.401)	(0.277)	–	–	–
CAR2_18_	–	–	–	–	–	–	−0.035	−0.027	−0.051
	–	–	–	–	–	–	(0.078)*	(0.222)	(0.640)
Tang	0.050	−0.001	0.745	0.046	−0.003	0.733	0.043	−0.005	0.734
	(0.339)	(0.988)	(0.008)***	(0.371)	(0.961)	(0.070)*	(0.401)	(0.915)	(0.010)***
Prof	−0.369	−0.328	0.781	−0.288	−0.287	0.826	−0.324	−0.286	0.679
	(0.017)**	(0.005)***	(0.346)	(0.063)*	(0.025)**	(0.298)	(0.036)**	(0.018)**	(0.420)
Size	−0.108	−0.021	−0.316	−0.106	−0.020	−0.314	−0.106	−0.020	−0.315
	(0.000)***	(0.021)**	(0.000)***	(0.000)***	(0.029)**	(0.000)***	(0.000)***	(0.038)**	(0.000)***
Lev	−0.094	0.042	−1.317	−0.103	0.036	−1.268	−0.094	0.039	−1.240
	(0.167)	(0.479)	(0.000)***	(0.121)	(0.527)	(0.016)**	(0.159)	(0.499)	(0.001)***
Const	1.889	0.552	6.101	1.863	0.539	6.040	1.854	0.525	6.055
	(0.000)***	(0.000)***	(0.000)***	(0.000)***	(0.000)***	(0.000)***	(0.000)***	(0.000)***	(0.000)***
N	165	165	165	165	165	165	165	165	165
Prob > F	0.000***	0.000***	0.015**	0.000***	0.000***	0.019**	0.000***	0.000***	0.017**
R^2^	0.586	0.081	0.352	0.599	0.088	0.347	0.594	0.093	0.344
Estimation with robust matrix	No	Yes	No	No	Yes	Yes	No	Yes	No
**Breusch-Pagan test**									
Chi^2^(1)	1.02	71.23	2.75	1.27	64.98	5.65	1.67	61.45	3.61
Prob > Chi^2^	0.314	0.000***	0.097*	0.260	0.000***	0.017**	0.197	0.000***	0.057*

Notes: *Cap1* is the amount of capital raised; *Cap2* is the number of shares issued; *Cap3* is the price per share. *CAR2*_*t*_ is accumulative abnormal return after share issue, where *t* = 6, 12 and 18 months. *Tang* is tangibility; *Prof* is profitability; *Size* is firm size; and *Lev* is book leverage. The variables *Tang*, *Prof*, *Size* and *Lev* were lagged by one quarter. We assumed significance of 5% in the Breusch-Pagan test for heteroscedasticity, i.e., when H_0_: homoscedasticity is rejected. The regression was performed by robust standard errors to correct the standard deviation for the possible presence of heteroscedasticity (White's correction). The table presents the linear coefficient of each explanatory variable followed by the p-value in parentheses. For rejection of the null hypothesis of coefficient equal to zero: ***significance at 1%; ** significance at 5%; * significance at 10%.

**Table 8:** **Effect of abnormal returns on primary share issues: total sample (IPOs and follow-on offerings), using the variable D_*CAR2***_***t***_**and sectorial market-to-book ratio (M/Bs)**. This table presents the data of cross-sectional regression. The complete sample was used in the analysis of the variable *D_CAR2*_*t*_ (*t* = 6, 12 and 18 months). The variables are those contained in Model 3, including the sectorial M/B (M/Bs) in place of the firm M/B, where *Y*_*ij*_ denotes the dependent variables *j Cap1*, *Cap2* and *Cap3*. Model 3.$Y_{ij}=\alpha +\beta_{1}\;{\left(D\_CAR2\right)}_{i}+\beta_{2}\;{\left(M/Bs\right)}_{i}+\beta_{3}\;{\left(Tang\right)}_{i}+\;\beta_{4}\;{\left(Prof\right)}_{i}+\;\beta_{5}\;{\left(Size\right)}_{i}+\beta_{6\;}{\left(Lev\right)}_{i}+u_{i}$.

	6 months			12 months			18 months		
	Cap1	Cap2	Cap3	Cap1	Cap2	Cap3	Cap1	Cap2	Cap3
D_CAR2_6_	0.034	0.016	0.189	–	–		–	–	
	(0.158)	(0.456)	(0.101)	–	–		–	–	
D_CAR2_12_	–	–		0.067	0.014	0.154	–	–	
	–	–		(0.006)***	(0.523)	(0.101)	–	–	
D_CAR2_18_	–	–		–	–		0.051	0.020	0.188
	–	–		–	–		(0.034)**	(0.366)	(0.055)*
M/Bs	0.016	−0.044	0.580	0.005	−0.047	0.551	0.010	−0.047	0.554
	(0.429)	(0.021)**	(0.012)**	(0.791)	(0.016)**	(0.017)**	(0.633)	(0.015)**	(0.016)**
Tang	0.042	0.021	0.468	0.043	0.021	0.470	0.042	0.021	0.470
	(0.425)	(0.669)	(0.096)*	(0.411)	(0.667)	(0.097)*	(0.417)	(0.666)	(0.091)*
Prof	−0.360	−0.280	0.339	−0.279	−0.271	0.420	−0.337	−0.274	0.364
	(0.020)**	(0.047)**	(0.698)	(0.072)*	(0.061)*	(0.641)	(0.028)**	(0.053)*	(0.689)
Size	−0.104	−0.029	−0.205	−0.107	−0.030	−0.215	−0.104	−0.029	−0.208
	(0.000)***	(0.000)***	(0.000)***	(0.000)***	(0.000)***	(0.000)***	(0.000)***	(0.000)***	(0.000)***
Lev	−0.077	0.001	−0.720	−0.092	−0.002	−0.747	−0.079	0.000	−0.724
	(0.268)	(0.992)	(0.040)**	(0.179)	(0.975)	(0.036)**	(0.250)	(0.999)	(0.038)**
Const	1.783	0.737	3.307	1.823	0.755	3.522	1.787	0.742	3.393
	(0.000)***	(0.000)***	(0.001)***	(0.000)***	(0.000)***	(0.000)***	(0.000)***	(0.000)***	(0.000)***
N	165	165	165	165	165	165	165	165	165
Prob > F	0.000***	0.000***	0.000***	0.000***	0.000***	0.000***	0.000***	0.000***	0.000***
R^2^	0.592	0.115	0.458	0.606	0.114	0.455	0.599	0.116	0.458
Estimation with robust matrix	No	No	Yes	No	No	Yes	No	No	Yes
**Breusch-Pagan test**									
Chi^2^(1)	0.7	1.27	126.45	0.46	2.23	134.11	0.11	2.72	127.95
Prob > Chi^2^	0.402	0.260	0.000***	0.497	0.135	0.000***	0.736	0.099*	0.000***

Notes: *Cap1* is the amount of capital raised; *Cap2* is the number of shares issued; *Cap3* is the price per share. D_*CAR2*_*it*_ assumes value 1 for firms that had negative cumulative abnormal return after the primary issue and 0 otherwise, in a time interval *t* of 6, 12 and 18 months. *M/Bs* is sectorial market-to-book ratio; *Tang* is tangibility; *Prof* is profitability; *Size* is firm size; and *Lev* is book leverage. The variables *Tang*, *Prof*, *Size* and *Lev* were lagged by one quarter. We assumed significance of 5% in the Breusch-Pagan test for heteroscedasticity, i.e., when H_0_: homoscedasticity is rejected. The regression was performed by robust standard errors to correct the standard deviation for the possible presence of heteroscedasticity (White's correction). The table presents the linear coefficient of each explanatory variable followed by the p-value in parentheses. For rejection of the null hypothesis of coefficient equal to zero: ***significance at 1%; ** significance at 5%; * significance at 10%.

**Table 9:** **Effect of abnormal returns on primary share issues: total sample (IPOs and follow-on offerings), using the variable D_*CAR2***_***t***_***,* without the variable market-to-book ratio (M/B)**. This table presents the data of cross-sectional regression. The complete sample was used in the analysis of the variable *D_CAR2*_*t*_ (*t* = 6, 12 and 18 months). The variables are those contained in Model 3, without the variable market-to-book ratio (M/B), where *Y*_*ij*_ denotes the dependent variables *j Cap1*, *Cap2* and *Cap3*. Model 3.$Y_{ij}=\alpha +\beta_{1}\;{\left(D\_CAR2\right)}_{i}+\beta_{2}\;{\left(Tang\right)}_{i}+\;\beta_{3}\;{\left(Prof\right)}_{i}+\;\beta_{4}\;{\left(Size\right)}_{i}+\beta_{5\;}{\left(Lev\right)}_{i}+u_{i}$.

	6 months			12 months			18 months		
	Cap1	Cap2	Cap3	Cap1	Cap2	Cap3	Cap1	Cap2	Cap3
D_CAR2_6_	0.033	0.018	0.166	–	–	–	–	–	–
	(0.152)	(0.413)	(0.175)	–	–	–	–	–	–
D_CAR2_12_	–	–		0.068	0.005	0.264	–	–	–
	–	–		(0.003)***	(0.825)	(0.033)**	–	–	–
D_CAR2_18_	–	–		–	–		0.052	0.013	0.266
	–	–		–	–		(0.028)**	(0.551)	(0.020)**
Tang	0.050	−0.001	0.744	0.045	−0.001	0.726	0.047	−0.001	0.730
	(0.354)	(0.990)	(0.064)*	(0.393)	(0.982)	(0.066)*	(0.358)	(0.977)	(0.062)*
Prof	−0.349	−0.312	0.758	−0.273	−0.322	1.013	−0.328	−0.317	0.866
	(0.069)*	(0.029)**	(0.314)	(0.154)	(0.027)**	(0.184)	(0.031)**	(0.027)**	(0.255)
Size	−0.107	−0.021	−0.313	−0.108	−0.021	−0.317	−0.106	−0.021	−0.307
	(0.000)**	(0.004)***	(0.000)**	(0.000)**	(0.003)***	(0.000)**	(0.000)**	(0.004)***	(0.000)**
Lev	−0.092	0.041	−1.249	−0.097	0.042	−1.266	−0.088	0.043	−1.232
	(0.275)	(0.506)	(0.017)**	(0.240)	(0.495)	(0.015)**	(0.181)	(0.487)	(0.016)**
Const	1.858	0.534	5.948	1.847	0.549	5.941	1.830	0.537	5.804
	(0.000)***	(0.000)***	(0.000)***	(0.000)***	(0.000)***	(0.000)***	(0.000)***	(0.000)***	(0.000)***
N	165	165	165	165	165	165	165	165	165
Prob > F	0.000***	0.000***	0.000***	0.000***	0.000***	0.000***	0.000***	0.000***	0.000***
R^2^	0.591	0.084	0.350	0.606	0.081	0.360	0.598	0.083	0.360
Estimation with robust matrix	Yes	No	Yes	Yes	No	Yes	No	No	Yes
**Breusch-Pagan test**									
Chi^2^(1)	4.93	0.73	67.83	4.42	2.05	76.9	2.77	2.24	70.37
Prob > Chi^2^	0.026**	0.392	0.000***	0.026**	0.152	0.000***	0.096*	0.134	0.000***

Notes: *Cap1* is the amount of capital raised; *Cap2* is the number of shares issued; *Cap3* is the price per share. D_*CAR2*_*it*_ assumes value 1 for firms that had a negative cumulative abnormal return after the primary issue and 0 otherwise, in a time interval *t* of 6, 12 and 18 months. *Tang* is tangibility; *Prof* is profitability; *Size* is firm size; and *Lev* is book leverage. The variables *Tang*, *Prof*, *Size* and *Lev* were lagged by one quarter. We assumed significance of 5% in the Breusch-Pagan test for heteroscedasticity, i.e., when H_0_: homoscedasticity is rejected. The regression was performed by robust standard errors to correct the standard deviation for the possible presence of heteroscedasticity (White's correction). The table presents the linear coefficient of each explanatory variable followed by the p-value in parentheses. For rejection of the null hypothesis of coefficient equal to zero: ***significance at 1%; ** significance at 5%; * significance at 10%.

**Table 10:** **Effect of abnormal returns on primary share issues: robustness analysis with an alternative stock market return index for the sample with 68 follow-on offerings**. This table presents the data of cross-sectional regression. Only the sample with 68 follow-on offerings was used in the analysis of the variable *CAR1*_*t*_ (*t* = 6, 12 and 18 months). *CAR1*_*t*_ was calculated by the benchmark-adjusted monthly returns method, for which the abnormal return is the difference between the firm's stock return and the average market return. The average market return is represented by the Brazil 100 Index (IBrX 100). The variables are those contained in Model 1, where *Y*_*ij*_ denotes the dependent variables *j Cap1*, *Cap2* and *Cap3*. Model 1.$Y_{ij}=\alpha +\beta_{1}\;{\left(CAR1\right)}_{i}+\beta_{2}\;{\left(M/B\right)}_{i}+\beta_{3}\;{\left(Tang\right)}_{i}+\;\beta_{4}\;{\left(Prof\right)}_{i}+\;\beta_{5}\;{\left(Size\right)}_{i}+\beta_{6\;}{\left(Lev\right)}_{i}+u_{i}$.

	6 months			12 months			18 months		
	Cap1	Cap2	Cap3	Cap1	Cap2	Cap3	Cap1	Cap2	Cap3
CAR1_6_	0.091	0.031	−0.021	**-**	**-**	**-**	**-**	**-**	**-**
	(0.006)***	(0.458)	(0.837)	–	–	–	–	–	–
CAR1_12_	**-**	**-**	**-**	0.072	0.013	0.032	**-**	**-**	**-**
	–	–	–	(0.008)***	(0.762)	(0.762)	–	–	–
CAR1_18_	**-**	**-**	**-**	**-**	**-**	**-**	0.026	−0.023	0.017
	–	–	–	–	–	–	(0.341)	(0.506)	(0.741)
M/B	0.014	−0.088	0.858	0.013	−0.089	0.860	0.010	−0.088	0.858
	(0.430)	(0.004)***	(0.000)***	(0.428)	(0.003)***	(0.000)***	(0.573)	(0.004)***	(0.000)***
Tang	0.090	0.117	0.072	0.095	0.119	0.070	0.096	0.119	0.070
	(0.012)**	(0.130)	(0.605)	(0.011)**	(0.121)	(0.766)	(0.011)**	(0.132)	(0.622)
Prof	−0.192	−0.324	−0.691	−0.188	−0.314	−0.727	−0.202	−0.246	−0.748
	(0.419)	(0.374)	(0.403)	(0.422)	(0.386)	(0.632)	(0.393)	(0.500)	(0.373)
Size	−0.021	−0.007	−0.009	−0.020	−0.009	0.000	−0.029	−0.016	−0.003
	(0.033)**	(0.630)	(0.803)	(0.040)**	(0.499)	(0.997)	(0.007)***	(0.238)	(0.925)
Lev	−0.113	0.066	−0.814	−0.136	0.052	−0.788	−0.153	0.035	−0.793
	(0.098)*	(0.459)	(0.049)**	(0.049)**	(0.560)	(0.052)*	(0.040)**	(0.700)	(0.039)**
Const	0.460	0.417	0.104	0.451	0.455	−0.059	0.613	0.571	−0.004
	(0.004)***	(0.108)	(0.889)	(0.006)***	(0.056)*	(0.945)	(0.001)***	(0.017)**	(0.995)
N	68	68	68	68	68	68	68	68	68
Prob > F	0.000***	0.010***	0.000***	0.000***	0.012**	0.000***	0.000***	0.009***	0.000***
R^2^	0.503	0.236	0.696	0.482	0.229	0.697	0.383	0.237	0.696
Estimation with robust matrix	Yes	Yes	Yes	Yes	Yes	No	Yes	Yes	Yes
**Breusch-Pagan test**									
Chi^2^(1)	5.85	8.04	4.06	5.52	8.01	3.82	11.03	11.12	3.98
Prob > Chi^2^	0.0155**	0.005***	0.044**	0.019**	0.005***	0.051*	0.001***	0.001***	0.046**

Notes: *Cap1* is the amount of capital raised; *Cap2* is the number of shares issued; *Cap3* is the price per share. *CAR1*_*t*_ is the cumulative abnormal return before share issue, where *t* = 6, 12 and 18 months. *M/B* is firm market-to-book ratio; *Tang* is tangibility; *Prof* is profitability; *Size* is firm size; and *Lev* is book leverage. The variables *Tang*, *Prof*, *Size* and *Lev* were lagged by one quarter. We assumed significance of 5% in the Breusch-Pagan test for heteroscedasticity, i.e., when H_0_: homoscedasticity is rejected. The regression was performed by robust standard errors to correct the standard deviation for the possible presence of heteroscedasticity (White's correction). The table presents the linear coefficient of each explanatory variable followed by the p-value in parentheses. For rejection of the null hypothesis of coefficient equal to zero: ***significance at 1%; ** significance at 5%; * significance at 10%.

**Table 11:** **Effect of abnormal returns on primary share issues: robustness analysis with an alternative stock market return index for the total sample, model with variable CAR2**_**t**_. This table presents the data of cross-sectional regression. The complete sample was used in the analysis of the variable *CAR2*_*t*_ (*t* = 6, 12 and 18 months). *CAR2*_*t*_ was calculated by the benchmark-adjusted monthly returns method, for which the abnormal return is the difference between the firm's stock return and the average market return. The average market return is represented by the Brazil 100 Index (IBrX 100). The variables are those contained in Model 2, where *Y*_*ij*_ denotes the dependent variables *j Cap1*, *Cap2* and *Cap3*. Model 2.$Y_{ij}=\alpha +\beta_{1}\;{\left(CAR2\right)}_{i}+\beta_{2}\;{\left(M/B\right)}_{i}+\beta_{3}\;{\left(Tang\right)}_{i}+\;\beta_{4}\;{\left(Prof\right)}_{i}+\;\beta_{5}\;{\left(Size\right)}_{i}+\beta_{6\;}{\left(Lev\right)}_{i}+u_{i}$.

	6 months			12 months			18 months		
	Cap1	Cap2	Cap3	Cap1	Cap2	Cap3	Cap1	Cap2	Cap3
CAR2_6_	−0.026	0.014	−0.489	–	–	–	–	–	–
	(0.555)	(0.750)	(0.012)**	–	–	–	–	–	–
CAR2_12_	–	–	–	−0.056	−0.027	−0.126	–	–	–
	–	–	–	(0.031)**	(0.389)	(0.277)	–	–	–
CAR2_18_	–	–	–	–	–	–	−0.032	−0.028	−0.027
	–	–	–	–	–	–	(0.110)	(0.190)	(0.763)
M/B	0.039	−0.083	0.770	0.037	−0.083	0.753	0.037	−0.083	0.754
	(0.036)**	(0.000)***	(0.000)***	(0.040)**	(0.000)***	(0.000)***	(0.041)**	(0.000)***	(0.000)***
Tang	0.028	0.046	0.313	0.025	0.045	0.308	0.023	0.042	0.310
	(0.596)	(0.325)	(0.170)	(0.626)	(0.340)	(0.184)	(0.663)	(0.380)	(0.184)
Prof	−0.421	−0.225	−0.206	−0.344	−0.175	−0.226	−0.382	−0.175	−0.383
	(0.007)***	(0.027)**	(0.757)	(0.028)**	(0.123)	(0.745)	(0.014)**	(0.093)*	(0.578)
Size	−0.098	−0.044	−0.108	−0.096	−0.043	−0.111	−0.096	−0.042	−0.113
	(0.000)***	(0.000)***	(0.007)***	(0.000)***	(0.000)***	(0.006)***	(0.000)***	(0.000)***	(0.005)***
Lev	−0.057	−0.034	−0.606	−0.067	−0.045	−0.533	−0.057	−0.042	−0.503
	(0.409)	(0.575)	(0.045)**	(0.328)	(0.449)	(0.081)*	(0.403)	(0.481)	(0.099)*
Const	1.662	1.037	1.592	1.644	1.023	1.644	1.638	1.009	1.673
	(0.000)***	(0.000)***	(0.016)**	(0.000)***	(0.000)***	(0.014)**	(0.000)***	(0.000)***	(0.014)**
N	165	165	165	165	165	165	165	165	165
Prob > F	0.000***	0.000***	0.000***	0.000***	0.000***	0.000***	0.000***	0.000***	0.000***
R^2^	0.598	0.215	0.592	0.608	0.221	0.578	0.603	0.227	0.575
Estimation with robust matrix	No	Yes	No	No	Yes	No	No	Yes	No
**Breusch-Pagan test**									
Chi^2^(1)	2.14	90.31	0.70	2.17	78.79	0.85	2.65	77.01	0.98
Prob > Chi^2^	0.147	0,000***	0.402	0.141	0.000***	0.358	0.104	0.000***	0.322

Notes: *Cap1* is the amount of capital raised; *Cap2* is the number of shares issued; *Cap3* is the price per share. *CAR2_t_* is the cumulative abnormal return after share issue, where *t* = 6, 12 and 18 months. *M/B* is firm market-to-book ratio; *Tang* is tangibility; *Prof* is profitability; *Size* is firm size; and *Lev* is book leverage. The variables *Tang*, *Prof*, *Size* and *Lev* were lagged by one quarter. We assumed significance of 5% in the Breusch-Pagan test for heteroscedasticity, i.e., when H_0_: homoscedasticity is rejected. The regression was performed by robust standard errors to correct the standard deviation for the possible presence of heteroscedasticity (White's correction). The table presents the linear coefficient of each explanatory variable followed by the p-value in parentheses. For rejection of the null hypothesis of coefficient equal to zero: ∗∗∗significance at 1%; ∗∗ significance at 5%; ∗ significance at 10%.

**Table 12:** **Effect of abnormal returns on primary share issues: robustness analysis with an alternative stock market return index for the total sample, model with variable D_CAR2**_**t**_. This table presents the data of cross-sectional regression. The complete sample was used in the analysis of the variable *D_CAR2*_*t*_ (*t* = 6, 12 and 18 months). *CAR2*_*t*_, which gave rise to the variable *D_CAR2*_t_, was calculated by the benchmark-adjusted monthly returns method, for which the abnormal return is the difference between the firm's stock return and the average market return. The average market return is represented by the Brazil 100 Index (IBrX 100). The variables are those contained in Model 3, where *Y*_*ij*_ denotes the dependent variables *j Cap1*, *Cap2* and *Cap3*. Model 3.$Y_{ij}=\alpha +\beta_{1}\;{\left(D\_CAR2\right)}_{i}+\beta_{2}\;{\left(M/B\right)}_{i}+\beta_{3}\;{\left(Tang\right)}_{i}+\;\beta_{4}\;{\left(Prof\right)}_{i}+\;\beta_{5}\;{\left(Size\right)}_{i}+\beta_{6\;}{\left(Lev\right)}_{i}+u_{i}$.

	6 months			12 months			18 months		
	Cap1	Cap2	Cap3	Cap1	Cap2	Cap3	Cap1	Cap2	Cap3
D_CAR2_6_	0.039	0.012	0.241	**-**	**-**	**-**	**-**	**-**	**-**
	(0.099)*	(0.569)	(0.024)**	–	–	–	–	–	–
D_CAR2_12_	**-**	**-**		0.073	0.027	0.167	**-**	**-**	**-**
	–	–		(0.002)***	(0.189)	(0.085)*	–	–	–
D_CAR2_18_	**-**	**-**	**-**	**-**	**-**		0.038	0.016	0.170
	–	–	–	–	–		(0.108)	(0.427)	(0.067)*
M/B	0.040	−0.082	0.767	0.037	−0.083	0.751	0.036	−0.084	0.744
	(0.030)**	(0.000)***	(0.000)***	(0.042)**	(0.000)***	(0.000)***	(0.052)*	(0.000)***	(0.000)***
Tang	0.030	0.047	0.328	0.029	0.047	0.317	0.029	0.047	0.322
	(0.562)	(0.304)	(0.323)	(0.570)	(0.329)	(0.340)	(0.571)	(0.303)	(0.332)
Prof	−0.393	−0.206	−0.183	−0.323	−0.178	−0.175	−0.391	−0.200	−0.239
	(0.011)**	(0.123)	(0.621)	(0.034)**	(0.080)*	(0.652)	(0.011)**	(0.135)	(0.576)
Size	−0.096	−0.043	−0.104	−0.098	−0.043	−0.114	−0.097	−0.043	−0.111
	(0.000)***	(0.000)***	(0.001)***	(0.000)***	(0.000)***	(0.000)***	(0.000)***	(0.000)***	(0.000)***
Lev	−0.060	−0.040	−0.551	−0.066	−0.043	−0.533	−0.057	−0.040	−0.523
	(0.380)	(0.504)	(0.220)	(0.320)	(0.481)	(0.239)	(0.405)	(0.506)	(0.241)
Const	1.619	1.020	1.401	1.626	1.019	1.601	1.638	1.022	1.565
	(0.000)***	(0.000)***	(0.007)***	(0.000)***	(0.000)***	(0.002)***	(0.000)***	(0.000)***	(0.002)***
N	165	165	165	165	165	165	165	165	165
Prob > F	0.000***	0.000***	0.000***	0.000***	0.000***	0.000***	0.000***	0.000***	0.000***
R^2^	0.604	0.216	0.589	0.620	0.223	0.582	0.603	0.217	0.582
Estimation with robust matrix	No	No	Yes	No	Yes	Yes	No	No	Yes
**Breusch-Pagan test**									
Chi^2^(1)	0.30	1.67	87.58	0.59	4.02	89.48	1.09	2.98	83.91
Prob > Chi^2^	0.584	0.196	0.000***	0.441	0.045**	0.000***	0.297	0.084*	0.000***

Notes: *Cap1* is the amount of capital raised; *Cap2* is the number of shares issued; *Cap3* is the price per share. D_*CAR2*_*it*_ assumes value 1 for firms that had a negative cumulative abnormal return after the primary issue and 0 otherwise, in a time interval *t* of 6, 12 and 18 months. *M/B* is firm market-to-book ratio; *Tang* is tangibility; *Prof* is profitability; *Size* is firm size; and *Lev* is book leverage. The variables *Tang*, *Prof*, *Size* and *Lev* were lagged by one quarter. We assumed significance of 5% in the Breusch-Pagan test for heteroscedasticity, i.e., when H_0_: homoscedasticity is rejected. The regression was performed by robust standard errors to correct the standard deviation for the possible presence of heteroscedasticity (White's correction). The table presents the linear coefficient of each explanatory variable followed by the p-value in parentheses. For rejection of the null hypothesis of coefficient equal to zero: ***significance at 1%; ** significance at 5%; * significance at 10%.

Finally, the dataset of the supplementary file (Excel spreadsheet) contains the following contents: Sheet 1: Description of dataset variables and sample selection (named “Data in Brief”); Sheet 2: Source of data; Sheet 3: Sub-Sample for CAR1 (only SEO); Sheet 4: Total Sample for CAR2 (IPO + SEO); Sheet 5: Sub-Sample (D_CAR1xD_CAR2), SEO; Sheet 6: Graphs.

## 2 Experimental design, materials, and methods

To calculate the abnormal return, we relied on the method employed by Ritter [2], of monthly benchmark-adjusted returns, for which the abnormal return is the difference between the firm's stock return and the average market return. To ascertain the abnormal returns before and after the share issue, each month was defined by 21 successive trading days in relation to the issue date (event). Under this setup, month 1 consists of days 1–21 after the event, month 2 includes days 22–42 after the event, and so on, until reaching days 169–189 (6th month), 232–252 (12th month), and 358–378 (18th month) post-event. We used the same method for abnormal returns before the share issue, but with month 1 composed of the 21 trading days before the event, until reaching the 6th, 12th and 18th month prior to the issue date (days 169–189, 232–252 and 358–378, respectively). We used the Bovespa Index (Ibovespa) and Brazil 100 Index (IBrX 100) to calculate the average market return, i.e., the benchmark.

In this study, the monthly abnormal returns were grouped in three different windows (−6, +6; −12, +12; −18, +18) by the cumulative average abnormal return (CAR) technique. Since we calculated the CAR per company, represented by only one asset (one stock), instead of a portfolio of assets, the CAR was adjusted only in the interval to reflect the abnormal return of 6, 12, and 18 months. To classify the firms with negative and positive abnormal returns, before and after the stock issue in the three different windows, we applied two dummy variables. The dummy *D_CAR1*_*t*_ assumes value 1 when a firm i had a positive cumulative abnormal return before share issue, and 0 otherwise, in a time interval t of 6, 12 or 18 months. In turn, the dummy *D_CAR2*_*t*_ assumes value 1 for firm i that had a negative cumulative abnormal return after share issue and 0 otherwise, in the same three intervals.

We used descriptive statistics and linear regression models to analyze the relationship of market timing and abnormal returns. We lagged the control variables by one period to minimize multicollinearity and problems of heteroscedasticity. We also used robust variance/covariance matrices of the parameters (White's correction) for the hypothesis of the existence of heteroscedasticity.

Table 2 shows the average values of the variables amount of capital raised (*Cap1*), number of shares issued (*Cap2*) and price per share (*Cap3*), segregated in the two groups of observations obtained by the measure *D_CAR1*_*t*_ for each of the three different windows (*t* = 6, 12 and 18 months), along with the difference of means test (Mann-Whitney). Since the sample analyzed was not normally distributed, we applied the nonparametric Mann-Whitney test, which permits comparing the means of independent samples extracted from the same population. The null hypothesis of this test is the absence of differences between the sampled groups [3]. We calculated the variables *Cap1*, *Cap2*, and *Cap3* according to the work of Alti [4]. Abnormal returns and cumulative abnormal returns were calculated by the benchmark-adjusted monthly returns following the method of Ritter [2], as commented before.

Table 3 shows the data of cross-sectional regression of Model 1 presented in Gomes et al. [1]. However, the variables are those contained in Model 1, including the sectorial M/B (*M/Bs*) in place of the firm M/B. Table 4 shows the data of cross-sectional regression of Model 1 but with the variable *M/B* deleted.

Similar to Table 1, Table 5 shows the average values of the variables *Cap1*, *Cap2* and *Cap3*, segregated in the two groups of observations, now obtained by the measure *D_CAR2*_*t*_, which assumes value 1 for firms with negative cumulative abnormal return after share issue and 0 otherwise, for each of the three different windows (*t* = 6, 12 and 18 months), along with the difference of means test (Mann-Whitney). The variables *Cap1*, *Cap2* and *Cap3* were calculated according to the work of Alti [4], while the abnormal returns were calculated following the method of Ritter [2].

Table 6 shows the data of cross-sectional regression of Model 2 presented in Gomes et al. [1]. However, the variables are those contained in Model 2, including the sectorial M/B (*M/Bs*) in place of the firm M/B. Table 7 shows the data of cross-sectional regression of Model 2 but with the variable *M/B* deleted.

Table 8 shows the data of cross-sectional regression of Model 3 presented in Gomes et al. [1]. However, the variables are those contained in Model 3, including the sectorial M/B (*M/Bs*) in place of the firm M/B. Table 9 shows the data of cross-sectional regression of Model 2 but with the variable *M/B* deleted.

Table 10, Table 11, Table 12 show the data of cross-sectional regression of Models 1, 2 and 3, respectively, with a change in the calculation of abnormal returns (*CAR1* and *CAR2*). Both *CAR1* and *CAR2* continue to be calculated by the benchmark-adjusted monthly returns method, for which the abnormal return is the difference between the firm's stock return and the average market return, but now the Brazil Index 100 (IBrX 100) is used instead of the Ibovespa to represent the average market return.

## Appendix A Supplementary data

Supplementary data to this article can be found online at www.tatianaalbanez.com/data or https://docs.wixstatic.com/ugd/4b053e_25c87bc429614a87a13387d7c334b58c.xlsx?dn=Data%20in%20Brief_Gomes%20et%20al.%20(2019).xlsx.
